# Single-leg cycling to maintain and improve function in healthy and clinical populations

**DOI:** 10.3389/fphys.2023.1105772

**Published:** 2023-04-28

**Authors:** C. Eric Heidorn, Steven J. Elmer, Kyle W. Wehmanen, James C. Martin, John McDaniel

**Affiliations:** ^1^ Vascular Health Lab, Exercise Physiology, Kent State University, Kent, OH, United States; ^2^ Louis Stokes Cleveland VA Medical Center, Cleveland, OH, United States; ^3^ Department of Kinesiology and Integrative Physiology, Michigan Technological University, Houghton, MI, United States; ^4^ Health Research Institute, Michigan Technological University, Houghton, MI, United States; ^5^ Department of Nutrition and Integrative Physiology, University of Utah, Salt Lake City, UT, United States

**Keywords:** modified exercise, one-leg cycling, blood flow, limb specific work, reduced muscle mass exercise

## Abstract

Exercise with reduced muscle mass facilitates greater muscle-specific adaptations than training with larger muscle mass. The smaller active muscle mass can demand a greater portion of cardiac output which allows muscle(s) to perform greater work and subsequently elicit robust physiological adaptations that improve health and fitness. One reduced active muscle mass exercise that can promote greater positive physiological adaptations is single-leg cycling (SLC). Specifically, SLC confines the cycling exercise to a smaller muscle mass resulting in greater limb specific blood flow (i.e., blood flow is no longer “shared” by both legs) which allows the individual to exercise at a greater limb specific intensity or for a longer duration. Numerous reports describing the use of SLC have established cardiovascular and/or metabolic benefits of this exercise modality for healthy adults, athletes, and individuals living with chronic diseases. SLC has served as a valuable research tool for understanding central and peripheral factors to phenomena such as oxygen uptake and exercise tolerance (i.e., V̇O_2peak_ and V̇O_2_ slow component). Together, these examples highlight the breadth of applications of SLC to promote, maintain, and study health. Accordingly, the purpose of this review was to describe: 1) acute physiological responses to SLC, 2) long-term adaptations to SLC in populations ranging from endurance athletes to middle aged adults, to individuals living with chronic disease (COPD, heart failure, organ transplant), and 3) various methods utilized to safely perform SLC. A discussion is also included on clinical application and exercise prescription of SLC for the maintenance and/or improvement of health.

## Introduction

Regular exercise is important for maintaining health across the lifespan, preventing and treating chronic disease, and enhancing athletic performance. The capacity to perform exercise, or exercise tolerance, can be attributed to central or peripheral factors within the integrated O_2_ transport system including but not limited to ventilation, alveolar-to-capillary oxygen diffusion, cardiac output, blood volume, peripheral vascular function, capillary density, oxidative enzyme concentrations, and muscle fiber type. The specific limiting factor(s) to exercise performance and peak oxygen consumption (V̇O_2peak_) can vary based on the health of the individual. While there is some debate regarding whether there is a central or peripheral limitation in healthy individuals (Bassett and Howley, 2000; Joyner and Dominelli, 2021), chronic obstructive pulmonary disease (COPD) and heart failure have historically been associated with central limitations and the individuals’ inability to deliver oxygenated blood to the working muscles (Martin et al., 1989; Richardson et al., 2004; Lee et al., 2016). Use of small muscle mass exercise (Richardson et al., 2004; Esposito et al., 2011; Lee et al., 2016) has facilitated separation of the influence of central cardiovascular and pulmonary dysfunction from peripheral muscle dysfunction. For example, isolated knee extension exercise completed in an aerobic fashion (e.g., 60 kicks/min for 15 min) is a small muscle mass exercise that has helped uncover the severe muscle dysfunction associated with COPD (Richardson et al., 2004) and heart failure (Esposito et al., 2011; Haykowsky et al., 2015). These findings should prompt reconsideration of rehabilitation strategies with these populations.

**FIGURE 1:**
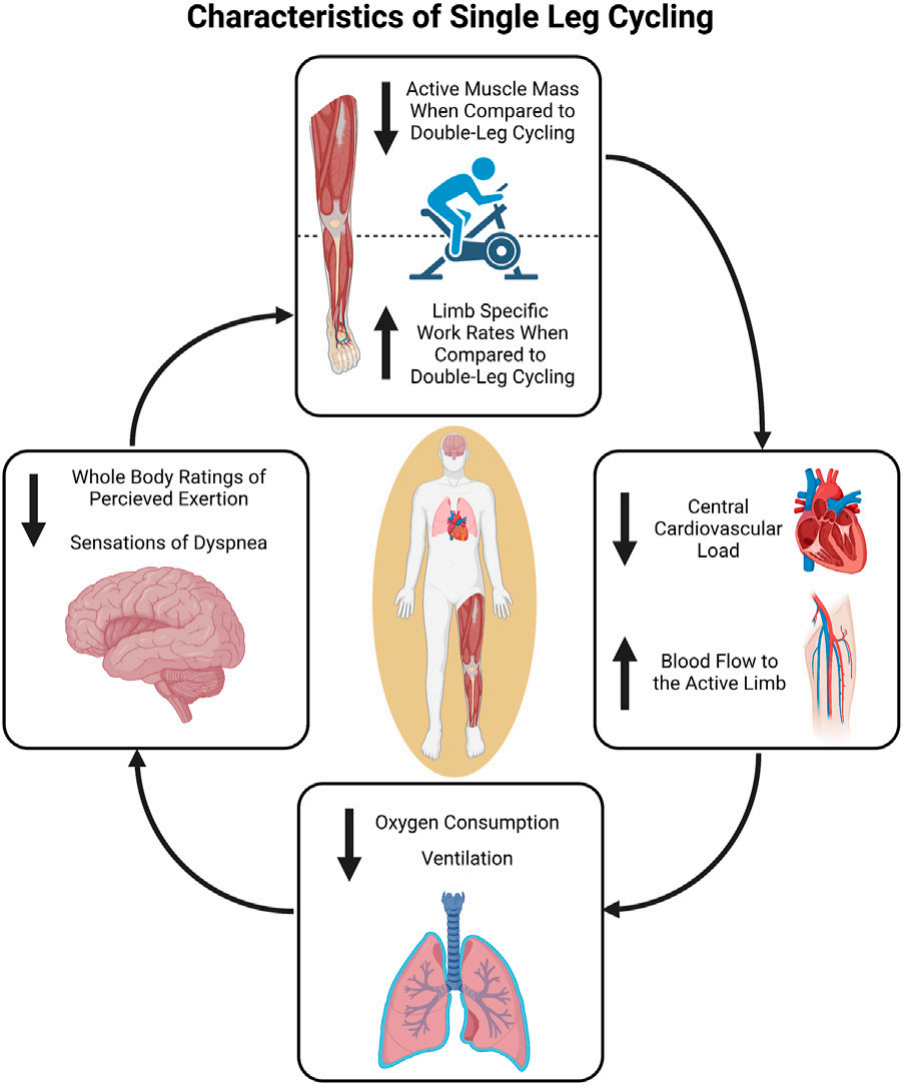
Overview of the physiological characteristics of single-leg cycling. The reduction in active muscle mass compared to double-leg cycling results in decreased central demands. This can in turn be utilized to facilitate comparatively greater limb specific work rates during single-leg cycling if central output is matched. Figure created in BioRender.com, with permission.

Another reduced muscle mass exercise is single-leg cycling (Figure 1). Specifically, single-leg cycling confines the exercise to a smaller muscle mass than traditional double-leg cycling resulting in greater limb specific blood flow (i.e., blood flow is no longer “shared” by both legs) (Burns et al., 2014b). This allows exercise at much greater limb specific intensity or for a longer duration at a similar limb specific intensity. The use of single-leg cycling stems from a report by Duner (1959) in which the author demonstrated that the working capacity of a single-leg was 73%–80% of both legs together and V̇O_2peak_ during single-leg cycling was approximately 91% of double-leg cycling. However, the use of single-leg cycling as a research tool has been limited, likely due to the fact that single-leg cycling is less tolerable and can be more difficult to coordinate (i.e., requires greater hip flexion to lift the leg) without cycle ergometer modifications that facilitate neuromuscular patterns similar to double-leg cycling (Burns et al., 2014a; Bini et al., 2015; Elmer et al., 2016). More recently, acute single-leg cycling has been used to study the differences in muscle perfusion (Burns et al., 2014b; LaScola et al., 2020), oxygen consumption (Draper et al., 2019; 2022), substrate utilization (Draper et al., 2019; Skattebo et al., 2022), and work capacity (McPhee et al., 2009; Gordon et al., 2018) between smaller muscle mass (single-leg cycling) and larger muscle mass exercise (double-leg cycling). Single-leg cycling has also been employed as an exercise training modality to maximize peripheral adaptations in both healthy and clinical populations (Dolmage and Goldstein, 2008; Bjørgen et al., 2009; Abbiss et al., 2011; Evans et al., 2015; Munch et al., 2018). In this review, the acute physiological responses and long-term adaptations to single-leg cycling are discussed for populations ranging from trained endurance athletes to individuals living with chronic disease. Various cycle ergometer modifications that could be used to safely perform single-leg cycling as well as the clinical application and exercise prescription of single-leg cycling for the maintenance and/or improvement of health are also presented in the current review.

## Methods

To assess the evidence supporting or refuting single-leg cycling as an efficacious exercise training and rehabilitation modality for both healthy and clinical populations, an electronic literature search was performed using the following keywords: single-leg cycling, one-leg cycling, single-limb cycling, and one-legged cycling as well as additional keywords from articles that were identified. Specifically, a search was completed for original articles published in peer-reviewed journals indexed on PubMed and Google Scholar between 1905-present. This search identified more than 100 articles. Many of these articles used single-leg cycling as a research intervention, but not directed towards understanding the acute and chronic adaptation to single-leg cycling as an exercise training modality. As a result, 16 and 11 articles were found that focused on the acute and chronic responses to single-leg cycle training, respectively. Thus, this review focuses on these 27 articles.

## Acute responses to single-leg cycling in healthy populations

Several investigators have used single-leg cycling to investigate and partition the central and peripheral physiological responses and limitations to exercise in aerobically trained and untrained healthy populations (McPhee et al., 2009; Burns et al., 2014b; MacInnis et al., 2017a; Gordon et al., 2018; 2020; LaScola et al., 2020) (Figure 1). When total work rate is matched between single- and double-leg cycling (i.e., 80 W) blood flow to the active leg during single-leg cycling has been reported to be 30%–90% greater than blood flow to the same leg during double-leg cycling (Burns et al., 2014b; LaScola et al., 2020). This is the case despite similar heart rate and blood pressure responses. At higher work rates (i.e., 120 W) single-leg cycling seems to elicit greater heart rate and blood pressure responses (Burns et al., 2014b; Skattebo et al., 2022) likely due to a greater limb specific metabolic demand and afferent feedback from the active muscle resulting in an increased sympathetic response.

The additional blood flow contribution to the active limb during single-leg cycling allows the individual to perform at a greater limb specific work rate (>50% of double-leg cycling) (Abbiss et al., 2011; Gordon et al., 2018; Skattebo et al., 2022). For example, power produced during 20 × 30 s intervals at a prescribed rating of perceived exertion of 15–17 with 60 s of recovery resulted in limb specific work rates of 99 ± 34 W during double-leg cycling and 134 ± 49 W during single-leg cycling (Gordon et al., 2019). Furthermore, single-leg cycling allowed for a 21% greater limb specific work rate (176 ± 52 W) during sequential 1 min intervals compared to double-leg cycling (145 ± 38 W) (Gordon et al., 2018). This increase in limb specific work rate was accomplished with reduced cardiovascular demand (26% lower exercising V̇O_2_ and 5% lower heart rate) (Gordon et al., 2018). Despite lower whole body oxygen demand during single-leg cycling, limb specific oxygen consumption is greater. McPhee and others (McPhee et al., 2009) reported that 76% (range of 58%–96%) of the V̇O_2peak_ achieved during double-leg cycling was consumed during single-leg cycling. This large range was attributed to variation in quadriceps muscle volume across individuals in that individuals with larger quadriceps volumes were able to achieve a greater percentage of the double-leg cycling V̇O_2peak_ during single-leg cycling. The greater limb specific intensity and limb specific V̇O_2_ reported by McPhee (McPhee et al., 2009) agrees with other reports (MacInnis et al., 2017a; Gordon et al., 2019; Skattebo et al., 2022). However, Gordon and others (Gordon et al., 2020) reported slightly greater cardiac output and oxygen consumption relative to active muscle mass during single-leg cycling compared to double-leg cycling. In this investigation, however, limb specific work rate during single-leg cycling did not exceed that achieved during double-leg cycling. This could mean greater familiarization periods for single-leg cycling may be necessary in this population or reflect the beginning of the decrease in local oxygen delivery and distribution capacity as commonly reported in the aging population (Dinenno et al., 1999; Black et al., 2009).

Manipulating limb specific intensity during single-leg cycling can elevate either glucose or fat oxidation compared double-leg cycling (Draper et al., 2019; Skattebo et al., 2022). When total work rate is equal between single- and double-leg cycling, the work rate for the active limb during single-leg cycling is twice that during double-leg cycling. This increased work rate results in greater respiratory exchange ratio and rate of glucose oxidation (Burns et al., 2014b; MacInnis et al., 2017a; Draper et al., 2019). While cycling at an intensity of 45% V̇O_2peak_, the increased carbohydrate oxidation during single-leg cycling compared to double-leg cycling (1.46 ± 0.45 vs. 1.01 ± 0.49 g/min) is associated with a reduction in fat oxidation (0.22 ± 0.06 vs. 0.36 ± 0.09 g/min) (Draper et al., 2019). This increase in glucose oxidation may acutely reduce blood glucose in those with diabetes and when performed over multiple sessions is likely responsible for increased GLUT-4 expression within the muscle (Abbiss et al., 2011). In contrast, during a ramp-protocol in which limb specific work rates were matched, mass specific maximal fat oxidation was 52% greater during single-leg cycling compared to double-leg cycling due to greater blood flow for any given muscle specific work rate (Skattebo et al., 2022). Together, these reports indicate single-leg cycling can be used to maximize peripheral utilization of either glucose or fatty acids.

Single-leg cycling may also offer benefits to athletes when training at altitude (Draper et al., 2019). Typically exercise capacity is attenuated at altitude due to reduced blood oxygen content and therefore oxygen delivery to the active muscle (Klausen et al., 1966; Dill and Adams, 1971; Gavin et al., 1998; Gatterer et al., 2021). Despite elevated blood flow to the muscle, the intensity of maximal effort single-leg cycling is also reduced at altitude (Gatterer et al., 2021). However, the reduction in tissue oxygenation is greater during double-leg cycling than single-leg cycling (Draper et al., 2022). Thus, it appears that the increase in blood flow to the active muscle during single-leg cycling may partially offset the reduced blood oxygen content and allow for higher limb specific training at altitude. Consequently, single-leg cycling could be utilized for athletes at altitude to replicate limb specific work rates that would be achieved at sea level. To summarize this section, single-leg cycling has been shown to result in greater limb specific blood flow to the active limb allowing for a greater limb specific work to be completed, enhanced limb specific substrate utilization, and possibly offset oxygen related decreases in training performance at altitude.

## Acute responses to single-leg cycling in clinical populations

The limited ability to ventilate the lungs and poor gas exchange within the pulmonary capillary network leads to reduced oxygen delivery to the active muscles resulting in exercise intolerance for individuals with pulmonary diseases such as COPD and pulmonary fibrosis (Dolmage and Goldstein, 2006; Dolmage et al., 2020). Greater blood flow to the reduced muscle mass during single-leg cycling should help offset the reduced blood oxygen content and improve either the duration or work rate of the exercising leg. Dolmage and Goldstein (2006) compared exercise capacity between single- and double-leg cycling in individuals with COPD (single-leg cycling work rate was set at half double-leg cycling work rate). Exercise tolerance was greater for the single-leg cycling condition as cycling with one leg allowed individuals with COPD to cycle 17 min longer and complete more total work in a single exercise session compared to double-leg cycling. In addition, during single-leg cycling participants had lower ventilation rates (-5.2 L/min), rating of dyspnea, and heart rate response (-9 bpm) at the end of exercise (Dolmage and Goldstein, 2006). A follow-up study (Dolmage et al., 2020) focused on the use of single-leg cycling in individuals with idiopathic pulmonary fibrosis during constant-load exercise and found similar improvements in endurance and work with lower V̇O_2_, ventilation, heart rate, and a higher tissue oxygen saturation at the end of single-leg cycling compared to double-leg cycling. These studies provide evidence that single-leg cycling allows for greater limb specific work rate and exercise tolerance in those individuals with pulmonary dysfunction.

Single-leg cycling has also been used with individuals with heart failure who have a well-established poor exercise tolerance due to reduced cardiac output, as well as poor peripheral vasculature and mitochondrial function (Bhella et al., 2011; Ives et al., 2016; Molina et al., 2016; Keir et al., 2021). LeJemtel et al. (1986) reported no difference in peak exercise leg blood flow between single and double-leg cycling in individuals with heart failure. Although this lack of increased blood flow during single-leg cycling could be due to the limited cardiac output and peripheral dilatory capacity, it is more likely due to subjects within this study achieving a greater limb specific work rate during double-leg cycling compared to single-leg cycling (LeJemtel et al., 1986). This could be due to the biomechanical constraints and hard to coordinate action of single-leg cycling on a non-modified cycle ergometer (see *Considerations for single-leg cycling ergometers* section for more details). Martin et al. (1989) reported no difference in maximal single- and double-leg cycling exercise responses for cardiac output, blood pressure, and a-vO_2_ difference in those with chronic heart failure. However, V̇O_2peak_ during single-leg cycling (1.15 ± 0.14 L/min) was well over half of that reported for double-leg cycling (1.33 ± 0.14 L/min) indicating greater metabolic demand of the active muscle during single-leg cycling (Martin et al., 1989). These data suggest that single-leg cycling may allow individuals to tolerate greater limb specific work rates which could result in improved peripheral adaptations for individuals with heart failure. Thus, single-leg cycling has been shown to improve limb specific blood flow, improve exercise tolerance, and allow for greater limb specific work to be completed in several clinical populations.

## Adaptations to single-leg cycling training in healthy populations

The adaptations to continuous or interval based single-leg cycle training has been examined in healthy and aerobically trained populations (Table 1). Single-leg cycling training has resulted in 6%–13% improvements in the trained leg V̇O_2peak_ and 8%–9% improvements in trained-leg work rate reached at the end of the incremental exercise test (Bell et al., 1988; Dela et al., 1993; Rud et al., 2012; MacInnis et al., 2017b). In addition, Rud and others (Rud et al., 2012) reported that the trained limb had a 30 ± 13% increase in citrate synthase activity, 4 ± 1% greater a-vO_2_ difference, and 21% ± 8% higher V̇O_2_ at a high-intensity work rate compared to the contralateral untrained limb. These adaptations, however, did not result in greater double-leg cycling V̇O_2peak_ suggesting that the adaptations were limb specific and/or there are central limitations to oxygen uptake during double-leg cycling (Rud et al., 2012). Dela and co-workers (Dela et al., 1993) investigated the use of 30 min of continuous single-leg cycling exercise at 70% V̇O_2peak_ in one leg 6 days a week for 10 weeks which resulted in a 26% increase in GLUT-4 protein concentration in the trained leg compared to the control leg.

**TABLE 1:** Adaptations to single-leg cycling training for healthy and clinical populations.

*Healthy Populations*					
Study	Population	Intervention	Frequency	Intensity	Key Findings
Bell et al. (1988)	Young adults	Exp: single-leg cycling HIIT	4x/wk for 7 weeks	15–20 × 20 s intervals	1-leg V̇O_2peak_ ↑ 6% in trained leg
	N = 9 (8 men 1 woman)	Con: no exercise in contralateral leg		Work to rest 1:3	2-leg V̇O_2peak_ ↑ 5%
				150% W_peak_	
Dela et al. (1993)	Young adults	Exp: single-leg cycling moderate continuous	6x/wk for 10 weeks	30 min	V̇O_2peak_ ↑ 13% in trained leg
					No change in V̇O_2peak_ of control leg
	N = 7 (7 men)	Con: no exercise in contralateral leg		70% 1-leg V̇O2_peak_	GLUT 4 protein concentration ↑26% in trained leg
					Greater increase compared to untrained leg
Rud et al. (2012)	Young adults	Exp: single-leg cycling moderate continuous	4x/wk for 7 weeks	40–100 min	V̇O_2peak_ ↑ 6% in trained leg
					No change in V̇O_2peak_ of control leg
	N = 12 (6 men 6 women)	Con: no exercise in contralateral leg		72% of 1-leg HR_peak_ (i.e., 108 W)	a-vO_2difference_ ↑ 4% in trained leg
					Greater citrate synthase activity in trained leg
MacInnis et al. (2017a)	Young adults	Exp1: single-leg cycling HIIT	3x/wk for 2 weeks	4 × 5 min intervals	No change in 1-leg V̇O_2peak_ for either group
				65% of 1-leg W_peak_ (i.e., 98 W)	W_peak_ ↑ 5%–9% after single-leg cycling HIIT and single-leg cycling continuous, no difference between trained legs
	N = 10 (10 men)	Exp2: single-leg cycling moderate continuous in contralateral leg		30 min	Greater citrate synthase activity after single-leg cycling HIIT trained leg
				50% of 1-leg W_peak_ (i.e., 75 W)	COX IV protein content ↑ after single-leg cycling HIIT and double-leg moderate continuous cycling, no difference between legs
Gordon et al. (2019)	Middle-aged adults	Exp: single-leg cycling HIIT, trained both legs, (n = 18)	3x/wk for 8 weeks	20 × 30 s intervals. 15–17 RPE with each leg (i.e., 130W)	2-leg V̇O_2peak_ and W_peak_ ↑ post-training, no difference between groups
	N = 53 (16 men, 37 women)	Con-1: double-leg cycling HIIT (n = 17)		20 × 30 s intervals 15–17 RPE (i.e., 199 W)	Resting blood pressure ↓ post-training, no difference between groups
		Con-2: double-leg cycling moderate continuous (n = 18)		40 min RPE 11–13 (i.e., 84 W)	Total cholesterol and LDL levels ↓post-training, no difference between groups
Abbiss et al. (2011)	Cyclists	Exp: single-leg cycling HIIT, trained both legs	2x/wk for 3 weeks	3 × 4 min intervals maximal intensity (i.e., 198 W)	No difference in 2-leg V̇O_2peak_ or time trial performance post-training
	N = 9 (9 men)	Con: double-leg cycling HIIT		3 × 4 min intervals maximal intensity (i.e., 344 W)	GLUT-4 protein concentration was greater after single-leg cycling HIIT
		6 weeks washout between conditions			COX II and IV protein content was greater after single-leg cycling HIIT

*Clinical Populations*					
Study	Population	Intervention	Frequency	Intensity	Key Findings
Bjørgen et al. (2009)	Individuals with COPD	Exp: single-leg cycling HIIT, trained both legs	3x/wk for 8 weeks	4 × 4 min intervals at 85%–90% 1-leg HR_peak_ rate alternating legs	2-leg V̇O_2peak_ ↑12% following single-leg cycling HIIT training, 6% following double-leg cycling HIIT, greater improvement following single-leg cycling HIIT
				4 × 4 min intervals at 85%–90% 2-leg HR_peak_	2-leg W_peak_ ↑23% following single-leg cycling HIIT and 12% following double-leg cycling HIIT, greater improvement following single-leg cycling HIIT
	N = 19 (7 men 12 women)	Con: double-leg cycling HIIT		Active recovery 3 min at 60%–70% 2-leg HR_peak_	1-leg V̇O_2peak_ and W_peak_ ↑ 18% and 37% following single-leg cycling HIIT, respectively
					1-leg peak ventilation ↑18% following single-leg cycling HIIT
Dolmage and Goldstein (2008)	Individuals with COPD	Exp: single-leg cycling moderate continuous	3x/wk for 7 weeks	30 min (15 min each leg) at highest tolerable work rate (∼40–60% W_peak_)	2-leg V̇O_2peak_ ↑ 22% following single-leg cycling
					Greater improvement compared to double-leg cycling
	N = 18 (8 men 10 women)	Con: double-leg cycling moderate continuous		30 min at highest tolerable work rate (∼55–70% W_peak_)	W_peak_ ↑ 35% following single-leg cycling
					Greater improvement compared to double-leg cycling
Evans et al. (2015)	Individuals with COPD	Exp: single-leg cycling moderate continuous	3x/wk for 6 weeks	15 min each leg at ∼40–70% W_peak_	2-leg V̇O_2peak_ ↑ 8%
	N = 22 (14 men 8 women)				2-leg peak power ↑ 17%
					6-min walk distance ↑ 23%
Munch et al. (2018)	Individuals with heart failure	Exp: single-leg cycling HIIT, trained both legs	3x/wk for 6 weeks	8 × 4 min intervals (4 intervals for each leg) at 90% 1-leg W_peak_	2-leg V̇O_2peak_ ↑ 8% relative and 14% absolute for participants with HF, 10% relative and 8% absolute for healthy participants
	N = 8 (7 men 1 women)				2-leg peak power ↑ 13% healthy, no change for participants with HF
	Healthy control			1.5–2 min rest between interval	1-leg peak knee extensor power ↑ 47% in participants with HF, no change in healthy participants
	N = 6 (5 men 1 women)				6-min walk distance ↑ 8% for individuals with HF, no change for healthy participants
del Torto et al. (2021)	Recipients of organ transplant	Exp: single-leg cycling HIIT, trained both legs (n = 17)	3x/wk for 8 weeks	12 sessions: 4 × 4 min intervals (work rate ≥ 5 RPE_leg fatigue_) 2 intervals each leg	2-leg V̇O_2peak_ ↑ 13% following single-leg cycling HIIT and 18% following double-leg cycling HIIT
	N = 33 (28 men, 5 women)			12 sessions: 6 × 2 min intervals (work rate ≥ 5 RPE_leg fatigue_) 3 intervals each leg	
	Heart (n = 13)			12 sessions: 4 × 4 min intervals (work rate ≥ 15 RPE_dyspnoea_) 3 min active recovery	
	Kidney (n = 11)	Con: double-leg cycling HIIT (n = 16)		12 sessions: 6 × 2 min intervals (work rate ≥ 15 RPE_dyspnoea_) 2 min active recovery	No difference between groups
	Liver (n = 9)				

Table 1 *Notes*: Exp, experimental condition; con, control condition; HIIT, high-intensity interval training; V̇O_2peak_, peak oxygen consumption; HR_peak_, peak heart rate; W_peak_, peak power reached during incremental V̇O_2peak_ test; RPE, rating of perceived exertion.

Not only are improvements seen in the active limb following single-leg cycling, but there is some indication that these improvements are greater than what is observed following standard double-leg cycling (Abbiss et al., 2011). Abbiss et al. (2011) reported that six sessions of single-leg cycling interval training in endurance trained cyclists resulted in much greater improvements in GLUT-4 and COX II and IV protein content compared to double-leg interval training. In addition, high-intensity single-leg cycling interval training is an equally effective method for reducing cardiovascular risk in middle-aged adults compared to moderate-intensity double-leg cycling (Gordon et al., 2019). Specifically, single-leg cycling resulted in improved cardiopulmonary fitness, resting systolic blood pressure, and circulating levels of total cholesterol and low-density lipoproteins (Gordon et al., 2019). Thus, single-leg cycling training has been reported to improve single leg V̇O_2peak_, citrate synthase activity, a-vO_2_ diff, GLUT-4 concentration, COX II and IV protein content, fitness, and to decrease cardiovascular risk factors, systolic blood pressure, and blood lipids in healthy populations.

## Adaptations to single-leg cycling training in clinical populations

The capability to maximize oxygen delivery to the working muscle during exercise can increase the magnitude of peripheral adaptations especially in those with more severe cardio pulmonary limitations. These limitations include a reduced maximum cardiac output or ventilation, poor gas exchange at the lungs, and reduced peripheral vascular function. Single-leg cycling has been utilized as a training modality for individuals with COPD (Dolmage and Goldstein, 2008; Bjørgen et al., 2009; Evans et al., 2015), heart failure (Munch et al., 2018), and organ transplants (i.e., heart, kidney, liver) (Del Torto et al., 2021; 2022). For example, Dolmage and Goldstein (2008) investigated the effects of individuals with COPD completing 30 min of either double-leg cycling or single-leg cycling (15 min per leg) exercise 3 days per week for 7 weeks. Single-leg cycling training resulted in a 21%, 13%, and 35% improvement in V̇O_2peak_, ventilation, and peak work rate, respectively. These improvements were greater than those observed following double-leg cycling. In related work, Bjørgen et al. (2009) investigated single-leg cycling in those individuals with COPD and compared 8 weeks of training 3 days per week completing 4 x 4-min bouts of either double-leg cycling (4 min active: 3 min active rest) or single-leg cycling (4 min alternating active legs) (Bjørgen et al., 2009). Single-leg cycling completed by individuals with COPD resulted in greater improvements in peak work rate (23% vs. 12%) and V̇O_2peak_ (12% vs. 6%) compared to double-leg cycling (Bjørgen et al., 2009). Finally, 6 weeks of high-intensity (8 × 4 min at 90% max work rate) single-leg cycling training completed by individuals with and without heart failure resulted in improvements in aerobic capacity and functional sympatholysis (Munch et al., 2018). An 8% improvement in 6-min walk test was also reported for individuals with heart failure that was not observed in those without heart failure (Munch et al., 2018). Collectively, these studies provide evidence that single-leg cycling has the capability to not only result in positive exercise adaptations and functional outcomes for individuals with chronic conditions but could generate greater improvements compared to traditional exercise methods such as double-leg cycling. As an additional note, a feasibility study on introducing single-leg cycling training in pulmonary rehabilitation clinics reported a high rate of training completion supporting single-leg cycling as a viable option to implement in clinical settings (Evans et al., 2015).

Most recently, single-leg cycling has been used by individuals with organ transplants. Following a transplant, severe deconditioning is observed which can largely be contributed to muscle dysfunction (Williams and McKenna, 2012) including metabolic dysfunction and muscle fiber type shifts (Kempeneers et al., 1990) as well as reductions in capillary density and endothelial dysfunction (Lampert et al., 1998; Braith et al., 2005). In addition, immunosuppressive drugs negatively affect skeletal muscle (Hokanson et al., 1995; Mercier et al., 1995). Thus, the potential for elevated limb specific work rate during single-leg cycling can potentially reverse these peripheral dysfunctions and improve overall exercise tolerance. The two reports indicated that single-leg cycling is as effective as double-leg cycling in improving V̇O_2peak_ (Del Torto et al., 2021) and V̇O_2_ kinetics (Del Torto et al., 2022) following 8-week of training in individuals with heart, lung, and kidney organ transplants. However, the specific training intensities (work rate) produced by the limbs during the training sessions is unclear as double-leg cycling intensity was set by dyspnea based rating of perceived exertion while intensity for single-leg cycling was based on leg fatigue rating of perceived exertion (Del Torto et al., 2021). Thus, single-leg cycling training has been shown to improve V̇O_2peak_ and kinetics, peripheral vascular function, functional performance such as walking, and the observed benefits may be greater than those following double-leg cycling training in various clinical populations.

## Other uses of single-leg cycling

Aside from utilizing single-leg cycling as a training modality to maximize peripheral adaptations, single-leg cycling has also served as an exercise option for individuals who cannot perform bilateral exercise and as a research tool to answer a variety of physiological questions. For example, individuals who have hemiplegia following a stroke or lower limb amputation may find it difficult to perform treadmill or cycling exercise, at least at the intensities required to improve cardiovascular health. The studies described above indicate that single-leg cycling can be used to achieve a cardiovascular and metabolic stress similar to double-leg cycling and therefore provides a valid exercise modality for individuals with hemiparalysis or lower limb amputation (Letombe et al., 2010; Wezenberg et al., 2012; Elmer and Martin, 2021) or during recovery from anterior cruciate ligament (ACL) injury (Olivier et al., 2008; 2010). Single-leg cycling has also been used to assess aerobic capacity between dominant and non-dominant limbs (Iannetta et al., 2019) and lower limbs with varied function due to multiple sclerosis (Larson et al., 2014) and ACL injury/surgery (Andrade et al., 2014; Hutchison et al., 2017; Bagley et al., 2020). As a research tool, single-leg cycling has been employed to partition central and peripheral contribution to exercise-induced fatigue (Elmer et al., 2013; Zhang et al., 2021), assess the impact of aerobic capacity on adaptations to resistance training (Thomas et al., 2022), evaluate recovery following eccentric muscle damage (Elmer et al., 2010; Gavin et al., 2015), investigate the influence of obesity on acute exercise response (Gries et al., 2022), and explore exercise muscle sympathetic nerve activity and the exercise pressor response (Stavres et al., 2020; Keir et al., 2021). Collectively, these examples highlight the breadth of applications of single-leg cycling to restore function, improve health, and facilitate research.

## Considerations for single-leg cycling ergometers

Implementation of single-leg cycling presents challenges. Most notably, the biomechanics of producing power during single-leg cycling are not the same as those during double-leg cycling. During double-leg cycling, net power is a combination of positive power produced during leg extension and negative power produced during leg flexion (Ericson, 1988; Neptune and Herzog, 1999; Elmer et al., 2011). With double-leg cycling the system is balanced in that the weight of the extending leg offsets the weight of the flexing leg requiring minimal recruitment of hip flexor muscles during the upstroke to produce a smooth cycling action. Conversely, with single-leg cycling the system is unbalanced, and the individual must actively lift the leg during the flexion phase (Elmer et al., 2016), which is unnatural and more difficult to coordinate resulting in greater perceived effort (Burns et al., 2014b) and reduced efficiency (Elmer and Martin, 2021). Thus, caution is needed when comparing physiological responses between single-and double-leg cycling with non-modified cycle ergometers (Neary and Wenger, 1986; Bell et al., 1988; Burns et al., 2014b) as the muscle recruitment is different between the two activities which could account for the differences in the reported physiological responses.

There have been several approaches to minimizing the abnormal cycling rhythm during single-leg cycling. For example, Gleser and others (Gleser, 1973) experimented with two different approaches: 1) springs attached to the active pedal to assist on the upstroke and 2) participants pedaling side-by-side with one foot of each subject secured to opposite pedals. Utilizing springs resulted in a non-linear relationship between V̇O_2_ and work rate while the side-by-side method maintained the linear V̇O_2_-workrate relationship traditionally observed with double-leg cycling and was therefore deemed to be more appropriate (Gleser, 1973). The use of a fixed gear cycle ergometer has also been utilized (Dolmage et al., 2014; Evans et al., 2015). Specifically, with this type of cycle ergometer, removal of the freewheel mechanism allows kinetic energy stored in the flywheel to help drive the single limb during hip flexion. Additionally, the use of a 10–11 kg ‘counterweight’ mounted to the unoccupied crank arm has been utilized during single-leg cycling (Burns et al., 2014b; Bini et al., 2015; Elmer et al., 2016; LaScola et al., 2020). This counterweight acts to help with the active leg during hip flexion but has no contribution to net power across an entire pedal cycle as that counterweight needs to be lifted during hip extension. Activation and work of hip and knee flexors is reduced when a counterweight is implemented, but it is still slightly greater than during double-leg cycling (Bini et al., 2015; Elmer et al., 2016). As a result, the counterweight reduces metabolic cost, cardiovascular demand, and perceived effort during single-leg cycling compared to when a counterweight is not utilized (Burns et al., 2014b; LaScola et al., 2020). Single-leg emphasis cycling facilitates cycling biomechanics of double-leg cycling without ergometer modifications. Simply encouraging participants to focus on “pushing down” with the emphasized leg during the downstroke and relax the de-emphasized leg, at least in trained speedskaters (who regularly performed cycling training), resulted in 75% of the power being produced by the emphasized leg and 25% being produced by the de-emphasized leg (Staples et al., 2020). Furthermore, the powerful extension action of the hip, knee, and ankle did not differ between single-leg emphasis cycling and double-leg cycling, but single-leg emphasis cycling required slightly more hip flexion (4%) (Staples et al., 2020). This could eliminate the need for a specialized ergometer and allow training to take place on traditional cycle ergometers and/or bicycles on the road but additional research is needed to support this. Interestingly, power-meter pedals are commercially available that can accurately quantify the work done by each leg when performing single-leg emphasis training. Finally, while assisted single-leg cycling (side-by-side, fixed gear, counterweight, emphasis) does not provide identical biomechanics to double-leg cycling (Bini et al., 2016; Elmer et al., 2016), it more closely resembles double-leg cycling than unassisted single-leg cycling.

## Implications and recommendations

The role of exercise intensity, duration, and volume in optimizing adaptations to rehabilitation and training has been pursued for decades. These questions also need to be answered for single-leg cycling and only a few studies have addressed these issues. As observed in Table 1 there is a range of training intensities across investigations that focus on long term adaptations to single-leg cycling in both healthy and clinical populations. The only study that directly compared how the intensity of single-leg cycling impacts the adaptations was by MacInnis and others (MacInnis et al., 2017b). In this investigation, participants performed high-intensity single-leg interval cycling in one leg with work matched moderate intensity single-leg cycling in the contralateral leg. Following only six sessions of training with each leg, citrate synthase activity and mass specific oxidative flux (phosphorylation capacities in complex I and complexes I and II) were greater in the limb that performed high-intensity interval cycling indicating a larger increase in mitochondrial volume (Larsen et al., 2012), compared to the limb that performed moderate intensity continuous cycling (MacInnis et al., 2017b). As a cautionary note, several of the authors of this review have performed extensive single-leg and single-leg-emphasis training and have, on occasion, experienced significant overtraining effects. This is likely due to the high metabolic stress that can be maintained during single-leg cycling and thus training volumes and intensities should error on the conservative side.

The impact of leg order during sequential single-leg cycling should also be considered during exercise prescription. Following single-leg cycling with the first leg, it is possible that the increased hyperemic response to single-leg cycling in the second leg could be less than the initial hyperemic response in the first leg. This seems to be the case following repeated maximal 60 s single-leg cycling efforts in which remaining vasodilation and blood pooling in the initial active limb reduces the redirection of blood flow to the subsequent active limb (Gordon et al., 2018). However, it is not clear if this would also be true following less intense bouts of single-leg cycling. Regardless, to gain maximal benefit of a single-leg cycling interval session, one should complete all intervals with the first leg before performing intervals on the second leg. Furthermore, it is recommended to alternate which leg performs the first bout of single-leg cycling between sessions.

**FIGURE 2:**
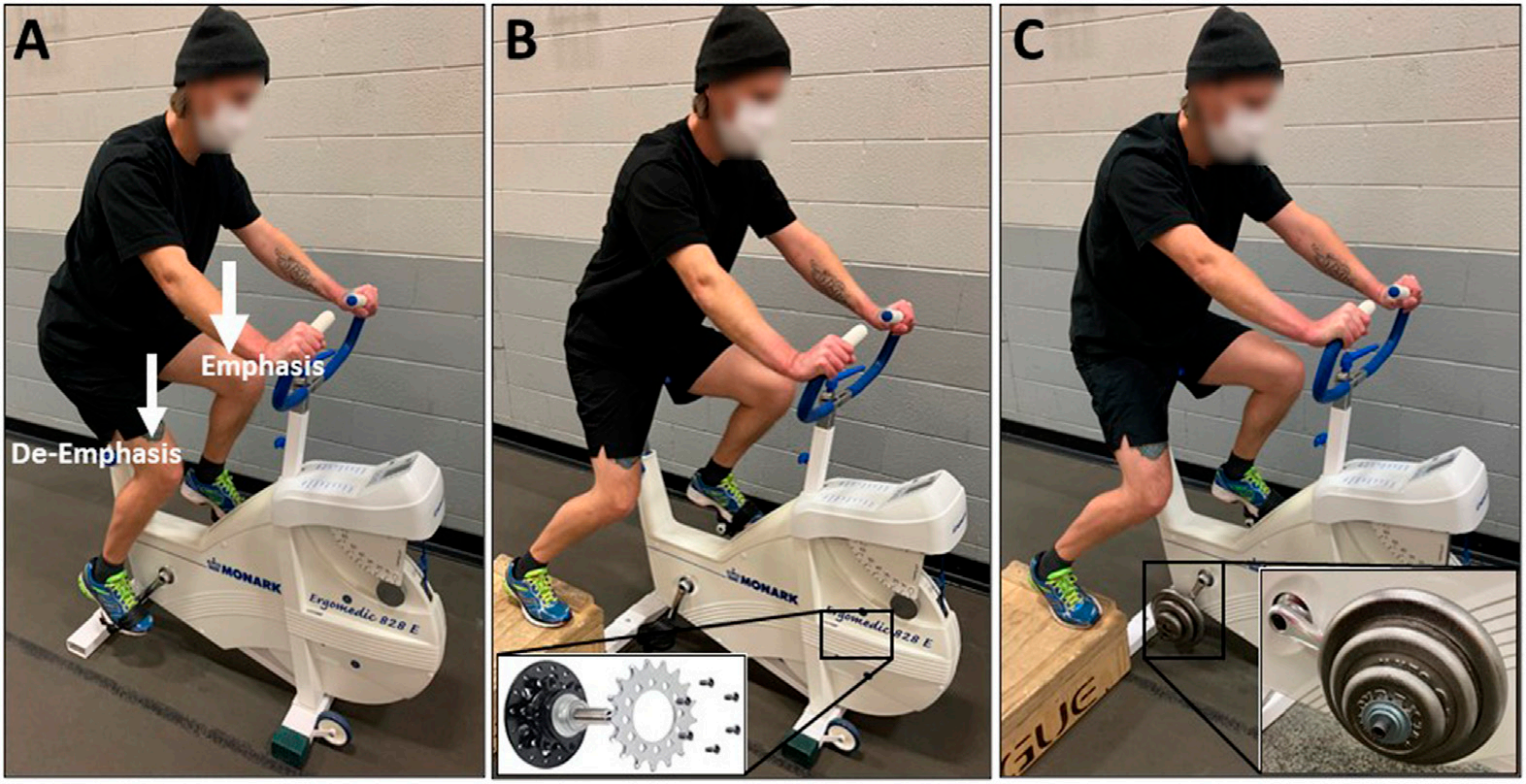
The various methods for facilitating single-leg cycling. **(A)** Single-leg emphasis: Using a standard cycle ergometer or bicycle, the individual cycles with both legs while instructed to focus on only one leg by “pushing down” during the limb extension phase. The contralateral leg is simply “along for the ride” and its weight helps during the flexion phase of the emphasized leg. **(B)** Fixed gear: The cycle ergometer is adapted by removing the freewheel mechanism from the drivetrain cog and affixing it directly to the flywheel. This allows the individual to cycle with one leg while utilizing the flywheel inertia to aid during the limb flexion phase. **(C)** Counterweight: The cycle ergometer is adapted by attaching a specialized pedal spindle with weights (∼10 kg) mounted to the contralateral crank arm. This allows the individual to cycle with one leg while utilizing the inertia of the counterweight to aid during the limb flexion phase.

Finally, here are some recommendations for using single-leg cycling. First, identify which single-leg cycling mode is available and if modifications to the ergometer are needed. The three most viable options are likely single-leg emphasis, fixed gear ergometer, and use of a counterweight (Figure 2). Second, familiarize the participant or patient with single-leg cycling so that the task can be performed safely and efficiently. Third, evaluate heart rate and rating of perceived exertion to ensure that the target single-leg cycling intensity is achieved. Fourth, complete all intervals with one leg first before performing intervals on the second leg. Fifth, alternate which leg performs the first bout of single-leg cycling between sessions. Finally, with any exercise intervention it is critical to monitor muscle soreness and joint pain to make sure that the exercise sessions are tolerated well over time.

## Summary

Reducing the active muscle mass during exercise by performing single-leg cycling decreases the cardiovascular demand and allows for an increased limb specific concentration of blood flow which can result in improved exercise tolerance and enhanced limb specific work rate. With chronic training, single-leg cycling can elicit cardiovascular and/or metabolic benefits in healthy adults, athletes, and individuals living with chronic diseases. Additionally, single-leg cycling serves as a viable exercise option for individuals who cannot perform bilateral exercise and as a research tool to answer a variety of physiological questions. This exercise modality is not without limitations, including the need for slight ergometer or pedaling (emphasis) modifications. Additional research is needed to better understand how to incorporate single-leg cycling into weekly exercise/training routines and determine the efficacy of single-leg cycling on performance in trained athletes and health in clinical populations.
